# Mechanical ventilation–associated AKI: toward a multimodal and kidney-protective ventilatory framework

**DOI:** 10.1186/s40635-026-00979-1

**Published:** 2026-10-16

**Authors:** Ying Gao, Yipeng Fang, Silvia De Rosa, Michele Salvagno, Lina Zhao, Keliang Xie, Fabio Silvio Taccone

**Affiliations:** 1 Department of Critical Care Medicine, Tianjin Medical University General Hospital, Tianjin, China; 2 Centre for Medical Sciences–CISMed, University of Trento, Trento, 38122 Italy; 3 Department of Intensive Care, Hôpital Universitaire de Bruxelles (HUB), Université Libre de Bruxelles, Brussels, Belgium; 4 Department of Anesthesiology, Tianjin Medical University General Hospital, Tianjin, China; 5 Department of Intensive Care, Erasme University Hospital - Université Libre de Bruxelles, Route de Lennik 808, Brussels, 1070 Belgium

**Keywords:** Mechanical ventilation, Acute kidney injury, Ventilation parameters, Renal perfusion, Lung–kidney cross talk

## Abstract

Mechanical ventilation–associated acute kidney injury (AKI) represents a complex manifestation of lung–kidney cross-talk driven by hemodynamic alterations, neurohumoral activation, systemic inflammation and disturbances in gas exchange and acid–base homeostasis. Beyond static ventilatory settings, increasing evidence suggests that dynamic fluctuations in airway and perfusion parameters, including static driving pressure (ΔP), positive end-expiratory pressure (PEEP), mean airway pressure (P_mean_), and effective renal perfusion pressure (eRPP) may substantially influence renal vulnerability during mechanical ventilation. This comprehensive narrative review synthesizes current mechanistic and clinical evidence on the relationship between mechanical ventilation and AKI and proposes an integrated framework for future research on the interplay between ventilatory stress, venous congestion, renal perfusion, and inflammatory injury. Emerging bedside tools such as renal Doppler indices, venous excess ultrasound score (VExUS), and kidney injury biomarkers are discussed as components of an integrated physiologic approach for early recognition and risk stratification of ventilator-associated AKI. We further propose a multimodal conceptual framework integrating respiratory mechanics, congestion assessment, perfusion monitoring, and biomarkers to support individualized kidney-protective ventilation strategies. Future research should prioritize standardized physiologic monitoring, time-varying exposure analyses, causal mediation approaches, and prospective multicenter studies to better define renal-protective ventilatory targets and advance precision critical care.

## Introduction

Acute kidney injury (AKI) is one of the most frequent complications in the intensive care unit (ICU), occurring in 30%–60% of patients receiving mechanical ventilation (MV), a rate substantially higher than in non-ventilated patients [1, 2]. Conversely, AKI increases the likelihood of acute respiratory failure requiring MV support [3]. The close lung–kidney interaction often results in the coexistence of both conditions, markedly worsening outcomes. Once AKI develops during MV, mortality rises significantly, accompanied by longer duration of ventilatory support, prolonged ICU stay, and greater need for renal replacement therapy (RRT) [4, 5].

Studies exploring the relationship between MV and AKI can be broadly categorized into mechanistic and clinical investigations. Experimental studies suggest that MV may contribute to AKI via interrelated hemodynamic, neurohumoral, inflammatory, and gas-exchange/acid–base pathways, which are summarized in the mechanistic section of this manuscript [6–8]. Clinically, growing evidence highlights the influence of key ventilatory parameters, in particular static driving pressure (ΔP) [9, 10] positive end-expiratory pressure (PEEP) [11–13], mean airway pressure (P_mean_) [14], and ventilation mode, [15] on renal outcomes. Bedside tools such as the renal resistive index (RRI), venous excess ultrasound score (VExUS), and biomarkers like urinary NGAL and the combination of TIMP-2 with IGFBP7 have also been investigated for early AKI detection and risk stratification [13, 15–17].

Nevertheless, most available studies are observational, limited by heterogeneity, inconsistent definitions, and inadequate causal inference. Integrated approaches that account for multiple mechanical and biological mechanisms remain scarce, limiting translation of experimental insights into practical bedside applications.

This review aims to provide a comprehensive synthesis of current evidence on how MV contributes to AKI, summarizing mechanistic insights, clinical findings, and emerging monitoring tools, and to identify methodological limitations and future directions toward the development of kidney-protective ventilation strategies. Beyond summarizing current evidence, this review proposes a multimodal conceptual framework integrating ventilatory exposure, renal perfusion, congestion assessment, and bedside monitoring strategies to support individualized kidney-protective ventilation.

## Literature search strategy

We conducted a structured literature search to identify mechanistic, experimental, and clinical studies addressing the relationship between mechanical ventilation and acute kidney injury. PubMed, Embase, and Web of Science were searched for articles published between January 2000 and September 2025. Search terms included combinations of “mechanical ventilation,” “ventilator-induced lung injury,” “positive end-expiratory pressure,” “driving pressure,” “mean airway pressure,” “acute kidney injury,” “renal perfusion,” and “lung–kidney cross-talk.” Reference lists of relevant reviews and original articles were also screened to identify additional studies.

We focused on peer-reviewed English-language original studies and major mechanistic or clinical reviews that provided mechanistic insight into lung–kidney interactions, evaluated associations between ventilatory parameters and renal outcomes, or investigated monitoring tools relevant to renal perfusion, venous congestion, or early AKI recognition. Given the heterogeneity of study designs, populations, ventilatory exposures, and renal outcomes, quantitative pooling was not performed. Instead, evidence was synthesized narratively and organized into mechanistic domains, clinical evidence, multimodal monitoring approaches, methodological limitations, and future research directions.

Most relevant clinical studies directly evaluating associations between ventilatory parameters and AKI-related outcomes are summarized in Table 2, whereas experimental studies and mechanistic reviews were used primarily to support the pathophysiological framework and future research agenda.

### Conceptual framework: lung–kidney cross talk during mechanical ventilation

The lungs and kidneys are tightly interrelated in maintaining systemic homeostasis. The respiratory system primarily regulates gas exchange and acid–base balance, whereas the kidneys maintain the internal milieu through water, electrolyte and acid–base regulation [18, 19]. In critical illness, this physiological coordination becomes disrupted, leading to complex bidirectional interactions, commonly referred to as lung–kidney cross talk.

In both physiological and pathological states, the lungs and kidneys interact through multiple interconnected pathways that link disturbances in respiration, circulation, and systemic inflammation with renal dysfunction. In ARDS or VILI, pulmonary injury alone can promote AKI via hypoxemia, systemic inflammation, and altered volume status, whereas AKI in turn worsens pulmonary function through fluid overload, toxin accumulation, and loss of cytoprotective mediators [7, 18–20]. Importantly, MV-associated AKI develops within a broader clinical context and may be modified by concurrent ICU interventions, including fluid management strategies (in particular a restrictive fluid approach), vasoactive therapy, and exposure to nephrotoxic medications. Therefore, the contribution of mechanical ventilation should be interpreted together with disease severity and other potentially modifiable iatrogenic factors. This reciprocal relationship forms the theoretical foundation for understanding how mechanical ventilation, by altering pulmonary and systemic physiology, contributes to the development of AKI.

**Fig. 1:**
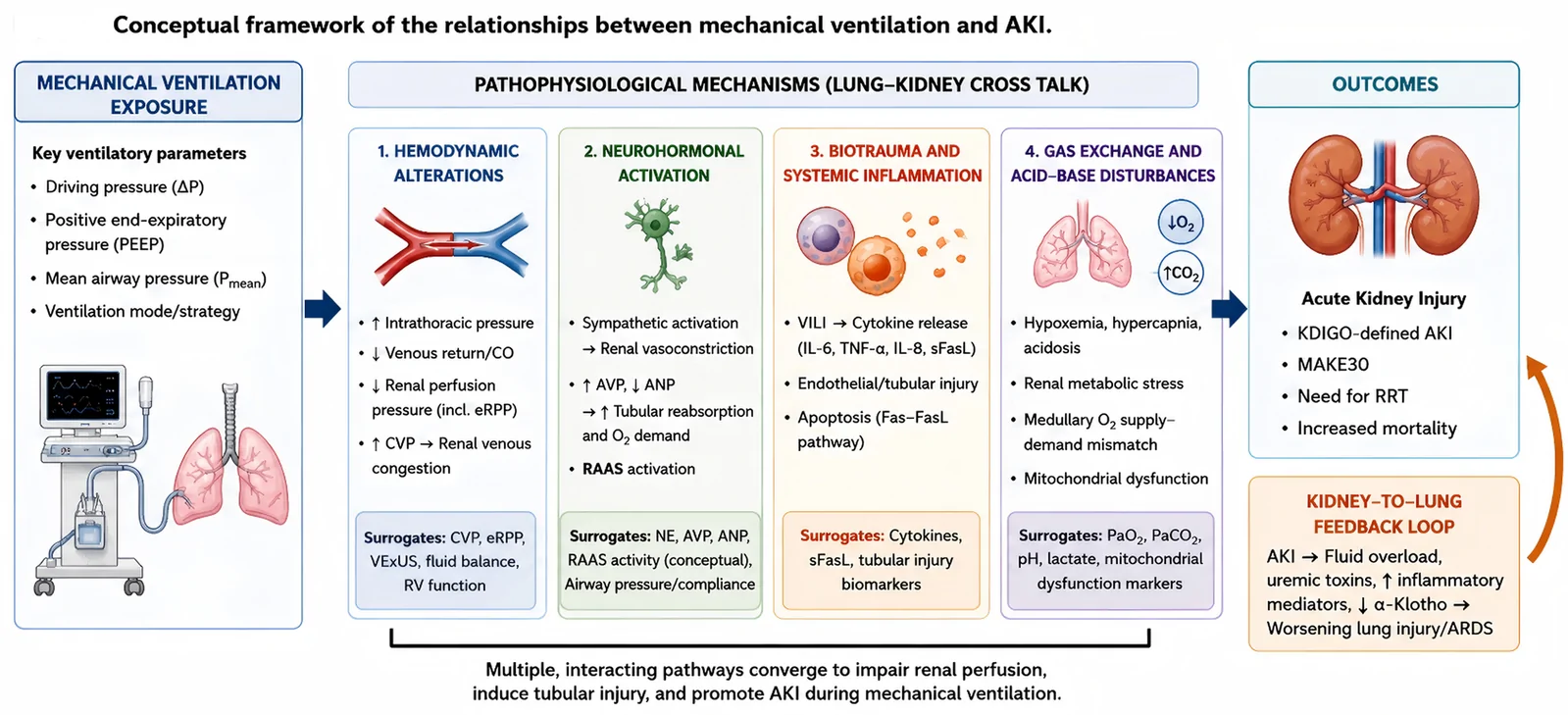
Conceptual framework linking mechanical ventilation to acute kidney injury. Mechanical ventilation exposures (ΔP, PEEP, Pmean, and ventilation mode/strategy) may contribute to AKI through four interacting mechanistic domains: (1) hemodynamic alterations (reduced venous return/cardiac output and increased venous congestion), (2) neurohormonal activation (sympathetic/RAAS/AVP–ANP imbalance), (3) biotrauma and systemic inflammation (cytokine release and endothelial/tubular injury), and (4) gas-exchange and acid–base disturbances (hypoxemia, hypercapnia, acidosis, and metabolic stress). AKI is associated with adverse outcomes (e.g., MAKE30, need for RRT, mortality) and may further worsen lung injury via a kidney-to-lung feedback loop, reinforcing lung–kidney cross-talk. Abbreviations: ΔP, driving pressure; PEEP, positive end-expiratory pressure; Pmean, mean airway pressure; AKI, acute kidney injury; CVP, central venous pressure; eRPP, effective renal perfusion pressure; VExUS, venous excess ultrasound score; RRI, renal resistive index; RAAS, renin–angiotensin–aldosterone system; AVP, arginine vasopressin; ANP, atrial natriuretic peptide; MAKE30, major adverse kidney events at 30 days; RRT, renal replacement therapy; ARDS, acute respiratory distress syndrome; VILI, ventilator-induced lung injury

We propose a conceptual framework linking key ventilatory exposures to AKI through four mechanistic domains and highlighting the kidney-to-lung feedback loop as part of cross-talk (Fig. 1).

### Mechanistic pathways linking mechanical ventilation to AKI

Building on this framework, ventilation-associated AKI can be understood across four major mechanistic domains: hemodynamic alterations, neurohormonal activation, biotrauma with systemic inflammation, and disturbances in gas exchange and acid-base homeostasis. The major pathophysiological mechanisms linking mechanical ventilation to AKI are summarized in Table 1.

**Table 1:** Summary of pathophysiological mechanisms linking mechanical ventilation to AKI

Mechanistic domain	Core physiological pathway	Key intermediate mediators / bedside surrogates	Representative evidence (as cited in review)	Practical implications (kidney-protective focus)
Hemodynamic alterations	Increased intrathoracic pressure reduces venous return and cardiac output, lowering renal perfusion pressure. PEEP-related increases in CVP impede renal venous drainage and raise interstitial pressure, promoting renal venous congestion	CVP; eRPP; fluid balance; VExUS; right ventricular dysfunction markers	20–23; clinical: 12,16	Avoid or mitigate venous congestion; hemodynamically guided PEEP adjustments; integrate perfusion and congestion assessment (not MAP alone)
Neurohormonal activation	MV-related sympathetic activation causes renal vasoconstriction and intrarenal blood flow redistribution. Increased AVP and reduced ANP promote sodium and water reabsorption and raise tubular oxygen demand, contributing to medullary hypoxia. RAAS activation reinforces a low-flow, high-reabsorption milieu	SNS/RAAS/AVP/ANP milieu; airway pressures; lung compliance; vasopressor requirement (context)	18,24; observational: 5	Recognize neurohumoral-hemodynamic coupling; interpret AKI risk in the context of airway pressures, compliance, and fluid/vasoactive strategy
Biotrauma and systemic inflammation	Injurious ventilation (high tidal volume/low PEEP; cyclic overdistension and collapse) promotes systemic spillover of inflammatory mediators (IL-6, TNF-alpha, IL-8, sFasL), leading to endothelial and tubular injury. Apoptosis pathways (Fas-FasL) may contribute	Circulating cytokines; sFasL; tubular injury biomarkers; CRRT for mediator removal/volume control (context)	25–27; 7	Reinforce lung-protective ventilation to reduce systemic biotrauma; consider inflammation and fluid balance jointly
Disturbances in gas exchange and acid-base homeostasis	Hypoxemia, hypercapnia, and respiratory acidosis increase renal metabolic stress. The medulla operates with relatively low perfusion but high metabolic demand, predisposing to oxygen supply-demand mismatch. In septic AKI, oxygen utilization is frequently inefficient, consistent with mitochondrial dysfunction and metabolic reprogramming	PaO2/PaCO2/pH; lactate (context); NGAL and TIMP-2·IGFBP7 as injury signals (context)	28–32	Avoid excessive gas-exchange derangements when feasible; interpret permissive hypercapnia with renal risk in mind; link acid-base status to renal metabolic stress
Kidney-to-lung feedback loop (bidirectional cross-talk)	AKI can worsen lung injury via volume overload and pulmonary edema, and through circulating uremic toxins, DAMPs, and inflammatory mediators that increase pulmonary permeability. Reduced alpha-Klotho may diminish pulmonary antioxidant and reparative capacity, aggravating ARDS	Fluid balance; extravascular lung water surrogates (context); inflammatory milieu; alpha-Klotho (conceptual)	33,34; 3,7,35	Recognize the vicious cycle; prioritize decongestion and balanced fluid strategy; integrate lung and kidney trajectories when evaluating MV course

AKI, acute kidney injury; ANP, atrial natriuretic peptide; AVP, arginine vasopressin; CVP, central venous pressure; DAMPs, damage-associated molecular patterns; eRPP, effective renal perfusion pressure; MV, mechanical ventilation; NGAL, neutrophil gelatinase-associated lipocalin; PEEP, positive end-expiratory pressure; RAAS, renin-angiotensin-aldosterone system; RRI, renal resistive index; sFasL, soluble Fas ligand; SNS, sympathetic nervous system; VExUS, venous excess ultrasound score

1. Hemodynamic alterations

Hemodynamic mechanisms play a pivotal role in this process. Positive-pressure ventilation increases intrathoracic pressure, which reduces right ventricular preload, venous return, and cardiac output, ultimately leading to a decline in renal perfusion pressure [21, 22]. Moreover, elevated positive end-expiratory pressure (PEEP) raises central venous pressure (CVP), impeding renal venous drainage and elevating interstitial pressure, which results in renal venous congestion [23]. Experimental ARDS models suggest that abrupt changes in PEEP, particularly under fluid overload, can aggravate venous congestion and contribute to combined lung–kidney injury, whereas hemodynamically guided PEEP adjustments may mitigate renal stress [24]. Clinical evidence further indicates that high CVP levels are independently associated with renal dysfunction [12, 16]. These hemodynamic changes reduce effective renal perfusion and predispose to AKI, particularly in patients with right heart dysfunction or fluid overload.

2. Neurohormonal activation

Mechanical ventilation (MV) elicits complex neurohumoral responses that profoundly influence renal perfusion and oxygen demand. Activation of the sympathetic nervous system during MV contributes to renal vasoconstriction and redistribution of intrarenal blood flow, thereby reducing oxygen delivery to vulnerable medullary regions18. Positive-pressure ventilation can also trigger non-osmotic release of arginine vasopressin (AVP/ADH) and suppression of atrial natriuretic peptide (ANP) secretion, shifting the kidney toward a state of enhanced water and sodium reabsorption, increased tubular oxygen consumption, and medullary hypoxia [25]. These hormonal disturbances, together with activation of the Renin–Angiotensin–Aldosterone System (RAAS) and sympathetic nervous system, may contribute to a state characterized by reduced renal oxygen delivery, enhanced sodium and water reabsorption, and increased metabolic demand in renal tubular cells. Observational data in invasively ventilated patients further support that elevated airway pressures and impaired lung compliance are associated with AKI incidence, suggesting that neurohumoral and hemodynamic coupling underlies the pathophysiology of lung–kidney cross-talk during MV5. Collectively, these findings indicate that sympathetic stimulation, AVP/ANP imbalance, and altered renal oxygen dynamics act synergistically to precipitate ventilator-associated AKI.

3. Biotrauma and systemic inflammation

Biotrauma and systemic inflammation represent another critical mechanism linking MV to AKI. Injurious ventilation, characterized by high tidal volume and low PEEP, can cause cyclic alveolar overdistension and collapse, triggering the release of proinflammatory cytokines such as IL-6, TNF-α, IL-8, and soluble Fas ligand (sFasL) into the systemic circulation. Compared with standard ventilation, lung-protective strategies significantly reduce these inflammatory mediators in both bronchoalveolar lavage and plasma and are associated with a lower incidence of AKI within 72 hours [19].

Experimental studies targeting inflammatory pathways have suggested potential renal-protective effects [26, 27]; however, translation of these approaches into clinical practice remains uncertain [19]. Continuous renal replacement therapy (CRRT) may facilitate fluid management in patients with AKI and respiratory failure; however, whether removal of circulating inflammatory mediators translates into clinically meaningful benefit remains uncertain [28]. Experimental data further demonstrate that injurious ventilation increases apoptosis in renal tubular and intestinal epithelial cells, while blocking the Fas–FasL pathway mitigates this damage [7]. Clinical evidence from lung-protective ventilation trials, particularly the ARMA trial, demonstrates that limiting injurious ventilatory stress improves clinical outcomes in ARDS [29]. Experimental studies further implicate soluble Fas ligand–mediated epithelial apoptosis as a potential pathway linking injurious ventilation to renal tubular injury [6, 7].

4. Disturbances in gas exchange and acid–base homeostasis

Gas-exchange abnormalities (hypoxemia and hypercapnia) and respiratory acidosis may increase renal metabolic stress and impair renal perfusion. The kidney is highly susceptible to hypoxia due to its unique oxygen metabolism and mitochondrial dependence [30]. Despite high oxygen consumption, renal blood flow distribution is heterogeneous, and the renal medulla operates at low perfusion but high metabolic demand, predisposing it to oxygen supply–demand mismatch [30, 31]. In septic AKI, renal oxygen delivery may be reduced or preserved, but oxygen utilization is frequently impaired, consistent with mitochondrial dysfunction [32]. Mitochondrial dysfunction further reduces ATP synthesis efficiency, suppresses fatty acid oxidation and glycolysis, and induces metabolic reprogramming that weakens tubular tolerance to hypoxia, rendering renal tubular cells extremely vulnerable [31]. Hypercapnia (i.e. PaCO₂ > 50 mmHg) impairs renal vasodilatory responses, reduces renal blood flow (RBF), and alters diuresis [33]. Respiratory acidosis increases oxygen consumption in proximal tubular cells, thereby exacerbating renal metabolic stress [34].

### Kidney-to-lung feedback loop

Although the focus here is MV-to-kidney injury, kidney dysfunction can also worsen lung injury, forming a vicious cycle. Impaired renal excretion leads to volume expansion and pulmonary fluid retention, resulting in pulmonary edema and worsening respiratory failure [35]. During AKI, uremic toxins, damage-associated molecular patterns (DAMPs), and inflammatory mediators circulate systemically, damaging alveolar epithelium and vascular endothelium and increasing pulmonary permeability. In sepsis models, resistin, an uremic toxin and pro-inflammatory cytokine, rises during AKI and impairs neutrophil bactericidal activity, linking kidney injury to pulmonary dysfunction [36]. In addition, AKI reduces α-Klotho expression, diminishing pulmonary antioxidant and reparative capacity and thereby aggravating ARDS [3, 7, 20].

Together, these pathways increase renal vulnerability during mechanical ventilation and provide the physiological rationale for interpreting clinical associations between ventilatory parameters and kidney outcomes.

### Clinical evidence linking ventilatory parameters to AKI

Clinical observational studies suggest that specific ventilatory parameters are associated with AKI risk and kidney-related outcomes, although RCTs with AKI as a primary endpoint remain limited. Table 2 summarizes selected clinical studies evaluating associations between ventilatory parameters and AKI-related outcomes.

**Table 2:** Clinical studies evaluating associations between ventilatory parameters and AKI-related outcomes in mechanically ventilated patients

Study (author, year)	Design / data source	Population (*N*)	Exposure (definition; window)	Outcome (definition; timing)	Main finding	Effect estimate (reported)	Key adjustment / notes
Andrianopoulos 2025	Observational study; ARDS cohort	ARDS; mechanically ventilated; N NR	Baseline driving pressure (ΔP) and mean ΔP over early ARDS course	Late AKI (timing after ARDS onset); KDIGO-based (details NR)	Higher ΔP independently associated with development of late AKI	aOR 1.046 (95% CI 1.021–1.072) per 1 cmH₂O; also OR 1.35 (1.15–1.58) per 1 SD = 6.6 cmH₂O	Adjusted for demographics, organ failures, fluid balance, PaO₂/FiO₂, RR, VT, Crs, PEEP
Casas-Aparicio 2025	Observational study; COVID-19 ARDS	COVID-19 ARDS; IMV; N NR	Early driving pressure (ΔP)	MAKE30; sustained AKI; biomarker-stratified	Higher early ΔP associated with higher MAKE30 risk; stronger signal when urinary NGAL > 40 ng/mL	NR	Effect modification by NGAL reported in paper
Dong 2025	Retrospective cohort; MIMIC-IV	Septic shock with MV; *n* = 956	Mean airway pressure (Pmean) trajectories (GBTM)	AKI occurrence; 30-day mortality	Higher/ascending Pmean trajectories associated with higher AKI risk; ~20.5% mediated by cumulative fluid balance	Trajectory groups 3–5 significant for AKI; mediation ACME 20.5% (95% CI 0.106–0.40)	Multivariable logistic/Cox; causal mediation analysis
Poirot 2025	Ancillary analysis; prospective multicenter COVID-ICU cohort	COVID-19 ARDS; IMV ≥ 48 h; *n* = 1,066	Daily mean PEEP averaged Days 1–3; continuous; threshold analysis	AKI within first 5 days after intubation (KDIGO)	Higher early PEEP independently associated with AKI; threshold ≈ 15.2 cmH₂O	OR 1.10 (95% CI 1.05–1.16) per 1 cmH₂O; threshold 15.2 cmH₂O	Sensitivity using KDIGO stage 2–3; imputation (MICE)
Valk 2021	Secondary analysis; PRoVENT-COVID multicenter observational	COVID-19 ARDS; invasively ventilated; *n* = 933	Higher vs. lower PEEP strategy (ARDSNet tables); first 4 days	AKI (KDIGO), RRT use, VFDs, mortality	Higher PEEP strategy associated with higher AKI incidence (matched) and more RRT use	NR	Propensity score matching; primary outcome VFDs
Leite 2019	Retrospective cohort with causal inference (DAG); MIMIC-III	ARDS; MV ≥ 48 h; VCV; *n* = 1,142	Time-weighted PEEP, ΔP, Pplat, VT/PBW, gases, compliance (first 48 h)	Severe AKI (KDIGO stage II–III) within 7 days	Only PEEP and compliance had direct causal association with severe AKI	PEEP OR 1.05 (95% CI 1.03–1.10) per 1 cmH₂O	Mediation analyses; nested CVP/PAP subcohorts
Basse 2023	Retrospective single-center cohort; COVID-19	COVID-19 ventilated; N NR	PEEP level; CVP; inflammation markers (as reported)	Renal dysfunction/acute renal failure (definition NR)	Higher PEEP associated with worse renal function with hemodynamic/inflammatory correlates	NR	Use as evidence of heterogeneity; avoid duplicating mechanism text
Fogagnolo 2024	Prospective single-center physiological study	Critically ill; MV expected ≥ 48 h; *n* = 92	PEEP trial 5/10/15 cmH₂O; RRI measured	AKI within 5 days (KDIGO)	RRI rises with PEEP; RRI ≥ 0.70 identifies higher AKI risk	AKI 55% vs. 13% (RRI ≥ 0.70 vs. < 0.70); AUROC 0.834 (mean RRI)	Supports individualized titration using renal Doppler
Yokoyama 2023	Retrospective analysis; Japanese ARDS registry (FORECAST ARDS)	ARDS; *n* = 151	Change in PEEP day4–day1 (ΔPEEP)	Worsening renal function (renal SOFA day1→day4)	Increased PEEP over days 1–4 associated with worsening renal function	OR 1.123 (95% CI 1.017–1.240) per 1 cmH₂O increase in ΔPEEP	Adjusted logistic and IPTW analyses
Cinotti 2020	Before–after practice change; single ICU (letter)	SARS-CoV-2 ventilated; *n* = 73	Period 1 higher PEEP + MAP > 65 vs. Period 2 PEEP limited (10–12) + MAP > 75 (first 48 h)	RRT by day 14; KDIGO stage by day 14	Practice change associated with reduced RRT use	RRT 45% vs. 21% (*p* = 0.038)	Observational; bundled intervention (PEEP + MAP+fluids)
Zacchetti 2024	Retrospective single-center observational; COVID-19 ARDS	COVID-19 ARDS on IMV; *n* = 144	Ventilatory/hemodynamic parameters (including plateau pressure); daily data until AKI	AKI within 15 days (KDIGO); RRT; 6-month mortality	Plateau pressure independently associated with AKI in multivariable model	NR	Includes fluid balance and diuretic dose; excludes missing CVP
Li 2024	Retrospective multicenter cohort	Elderly ≥ 75 yrs; IMV; *n* = 1,292	PEEP at MV start; oxygenation; labs	Early AKI ≤ 48 h vs. late AKI > 48 h–7 d; mortality	PEEP at MV initiation associated with early AKI (not late); early/late AKI associated with higher mortality	28-day mortality: 14.4% vs. 46.8% vs. 61.4%; HR early 4.035, late 6.272 (28-day)	Highlights timing heterogeneity relevant to causality
Perschinka 2025	Registry-based propensity-matched analysis (Austria)	Non-surgical ICU > 48 h; matched COVID vs. control; 4,339 per group (total 8,678)	Invasive mechanical ventilation (time-dependent)	RRT initiation (time-to-event); ICU mortality (RRT subgroup)	IMV associated with increased risk of RRT initiation (stronger in COVID); IMV among RRT patients linked to higher ICU death risk	NR (time-split HRs)	Ventilation exposure vs. parameter level
Vemuri 2022	Retrospective cohort; UC San Diego ICUs	Adult ICU admissions; *n* = 9,704; IMV *n* = 4,484	AKI during IMV (KDIGO) vs. no AKI; mechanics summarized during IMV	ICU mortality; ventilator days; ICU LOS; respiratory mechanics	AKI during IMV associated with higher mortality and worse mechanics (higher Pplat/ΔP; lower compliance)	Adjusted mortality hazard 1.58 (95% CI 1.22–2.03)	Mechanics differences persisted even with net negative fluid balance
Kopitko 2018	Prospective multicenter postoperative monitoring study	Major abdominal surgery; *n* = 84	Renal perfusion pressure equations incorporating MAP, CVP, IAP, Pmean	AKI at 48 h (AKIN)	Composite perfusion pressure equations outperformed MAP alone for AKI prediction	Best-performing equation AUC ~ 0.791 (reported)	Supports eRPP concept integrating intrathoracic factors
Kopitkó 2019	Prospective observational; major abdominal surgery	Major abdominal surgery; *n* = 84 (subset ventilated)	Effective RPP = MAP − (IAP + CVP + Pmean); admission/first 12 h	AKI at 48 h (AKIN)	Effective RPP predicted AKI; threshold ~ 40.7 mmHg at admission linked to universal AKI	Threshold ~ 40.7 mmHg (reported)	Time-sensitive target; relevant to early postoperative period

NR, not reported; AKI, acute kidney injury; ΔP, driving pressure; PEEP, positive end-expiratory pressure; Pmean, mean airway pressure; Pplat, plateau pressure; RRT, renal replacement therapy; VFDs, ventilator-free days; CVP, central venous pressure; IAP, intra-abdominal pressure; eRPP/RPP, effective renal perfusion pressure; RRI, renal resistive index; VExUS, venous excess ultrasound score; ARDS, acute respiratory distress syndrome; COVID-19, coronavirus disease 2019; IMV, invasive mechanical ventilation; PBW, predicted body weight; Crs, respiratory system compliance

Among ventilatory variables, ΔP has one of the most consistent associations with AKI-related outcomes. Multiple observational studies and an individual patient data meta-analysis have shown that elevated ΔP is independently associated with AKI and major adverse kidney events (MAKEs) [9, 10]. In patients with SARS-CoV-2–related ARDS receiving invasive mechanical ventilation, higher early driving pressure was independently associated with an increased risk of major adverse kidney events at 30 days (MAKE30). In the same cohort, urinary NGAL levels above 40 ng/mL further helped identify patients at increased risk of sustained AKI [10], supporting the potential value of combining ventilatory parameters with kidney injury biomarkers for risk stratification.

The role of PEEP remains controversial. Experimental studies suggest that high PEEP may increase AKI risk in the setting of elevated mechanical power [9]. Clinically, findings are heterogeneous: some cohorts report an association between higher PEEP and renal deterioration [11, 16], whereas titration studies highlight marked interindividual variability. In a prospective study, an increase in RRI to ≥ 0.70 during PEEP titration identified patients with a substantially higher subsequent AKI incidence [13], supporting an individualized approach in which renal tolerance to PEEP is assessed dynamically rather than inferred from PEEP level alone.

Mean airway pressure (P_mean_), which integrates both respiratory and circulatory load, has recently gained recognition as a comprehensive indicator of ventilatory burden. A large trajectory analysis using the MIMIC-IV database found that persistently high P_mean_ was strongly associated with increased mortality and AKI, with the highest AKI incidence observed in the high–stable trajectory group [14]. In this study, positive fluid balance explained about 20% of the P_mean_–AKI relationship, highlighting hemodynamic–respiratory mediation as a potential mechanism.

The mode of ventilation may also influence renal perfusion and outcomes. A recent viewpoint proposed the “venous bagging” hypothesis, suggesting that controlled mechanical ventilation (CMV), by abolishing the diaphragm’s negative pressure pump, predisposes to venous congestion and AKI, whereas pressure-supported ventilation (PSV) may improve venous return and reduce AKI incidence [15]. Observational data from COVID-19 cohorts similarly indicated a lower AKI incidence among patients managed with early spontaneous breathing support [15].

Overall, ΔP and Pmean show the most consistent associations with kidney outcomes, whereas the effects of PEEP and ventilation mode appear more context-dependent and influenced by individual hemodynamics and venous congestion.

### Recognition and prediction of AKI during mechanical ventilation

Accurate recognition of renal hypoperfusion and early identification of AKI in mechanically ventilated patients remain major clinical challenges [17]. Traditional hemodynamic targets such as mean arterial pressure (MAP) alone are insufficient to represent true renal perfusion, as they fail to account for venous congestion and intrathoracic pressure effects [20]. To address this limitation, effective renal perfusion pressure (eRPP = MAP − [CVP + IAP + P_mean_], where central venous pressure = CVP; intra-abdominal pressure = IAP and mean airway pressure = P_mean_) has been described in studies integrating arterial pressure with other relevant variables [37, 38]. By integrating the impact of CVP, IAP and P_mean_, eRPP better reflects the net driving force of renal blood flow [14, 17]. Clinical observations have shown that lower eRPP values are more predictive of AKI than MAP alone, emphasizing the combined influence of both arterial inflow and venous outflow determinants [2]. Kopitko et al. advanced this concept through sequential studies; a protective eRPP threshold of approximately 40 mmHg, below which postoperative AKI was almost universal, was identified [37, 38].

Bedside ultrasonography is emerging as a valuable non-invasive tool for assessing renal perfusion. The RRI, derived from Doppler ultrasonography, quantifies intrarenal vascular resistance and has demonstrated predictive value for AKI in mechanically ventilated patients. In a prospective study, an elevated RRI during PEEP titration independently predicted the occurrence of AKI, suggesting that real-time renal Doppler assessment may guide individualized ventilator adjustments [13]. Complementarily, the VExUS integrates venous Doppler findings from the hepatic, portal, and renal veins to quantify systemic venous congestion [16]. Although clinical validation remains limited, emerging evidence indicates that higher VExUS grades correlate with venous congestion–related AKI and may enhance bedside evaluation of renal hemodynamics.

In addition to physiologic monitoring, biomarkers and predictive modeling are being explored to improve early detection and risk stratification of MV-associated AKI. Urinary neutrophil gelatinase–associated lipocalin (NGAL) has been identified as an independent predictor, with concentrations > 40 ng/mL associated with persistent AKI in critically ill patients (adjusted OR of 8.54) [10]. Other biomarkers, such as IL-6 and TIMP-2 with IGFBP7 show promise for early AKI prediction but remain inconsistent across studies. Integrating these biological indicators with ventilatory and hemodynamic parameters could improve the performance of risk prediction models. Future research should validate these multimodal frameworks and integrate them into individualized ventilation strategies to achieve kidney-protective critical care.

**Fig. 2:**
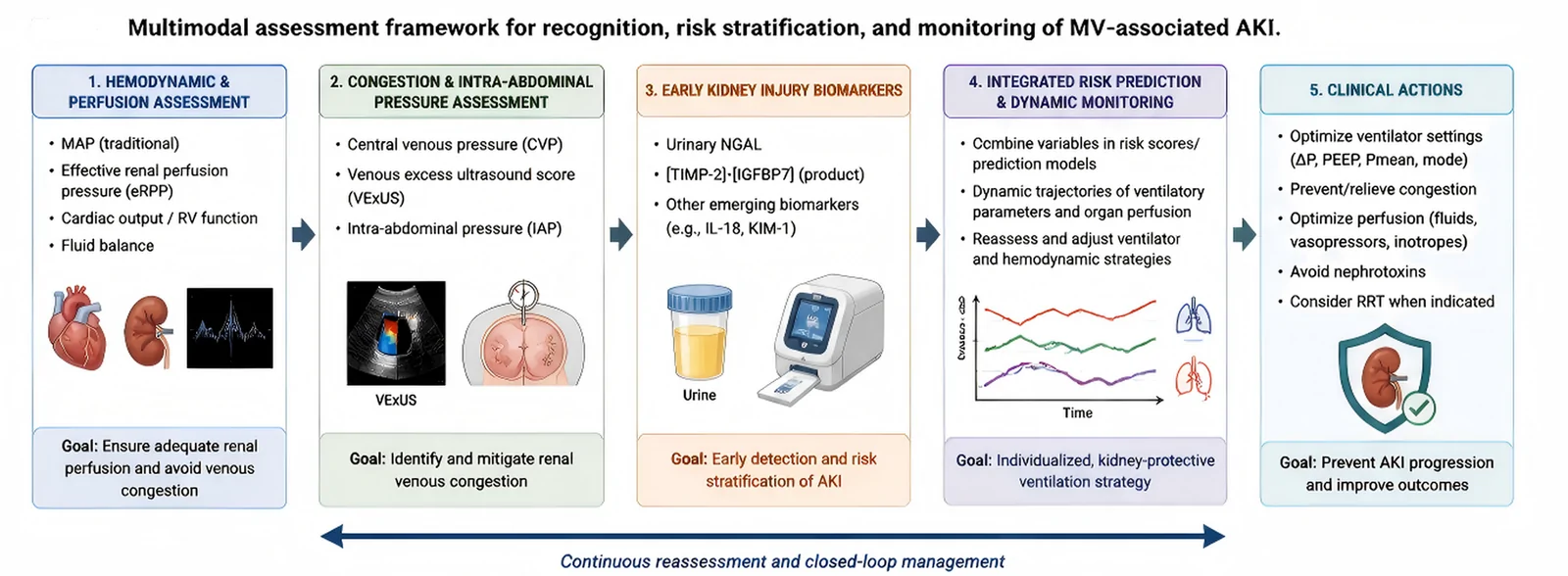
Multimodal assessment framework for recognition, risk stratification, and monitoring of MV-associated AKI. Proposed stepwise bedside framework integrating (1) hemodynamic/perfusion assessment (including eRPP), (2) venous congestion and intra-abdominal pressure assessment (CVP, VExUS, IAP), (3) early kidney injury biomarkers (e.g., urinary NGAL, TIMP-2·IGFBP7), and (4) integrated risk prediction with dynamic monitoring of time-varying ventilatory and perfusion trajectories (ΔP, Pmean, eRPP). These inputs support (5) clinical actions aimed at kidney-protective ventilation, including optimization of ventilator settings and perfusion while preventing or relieving congestion, with continuous reassessment in a closed-loop manner.Abbreviations: MV, mechanical ventilation; AKI, acute kidney injury; MAP, mean arterial pressure; eRPP, effective renal perfusion pressure; CVP, central venous pressure; IAP, intra-abdominal pressure; Pmean, mean airway pressure; ΔP, driving pressure; PEEP, positive end-expiratory pressure; VExUS, venous excess ultrasound score; RRI, renal resistive index; NGAL, neutrophil gelatinase-associated lipocalin

To translate these physiological concepts into bedside practice, we propose a multimodal assessment framework integrating perfusion, congestion, biomarkers, and time-varying ventilatory exposure for AKI recognition, risk stratification, and iterative management (Fig. 2). Although bedside implementation currently relies mainly on measurable physiological variables, neurohumoral activation and inflammatory pathways represent important biological layers that may be incorporated through circulating biomarkers or mechanistic mediators in future multimodal models.

## Future research directions

### Time-varying exposure and trajectory-based analyses

Most studies have evaluated ventilatory variables or hemodynamic indices using single time-point measurements. However, the temporal trajectory of these variables may better capture the exposure-response relationship with renal dysfunction. Continuous or time-weighted monitoring of driving pressure, P_mean_, and eRPP could reveal early warning thresholds associated with AKI onset. Similarly, metrics reflecting renal perfusion pressure, such as renal mean perfusion pressure (MPP), have shown prognostic value in critically ill patients with AKI. In a large MIMIC-IV–based study of sepsis-associated AKI, Fang et al. identified distinct dynamic MPP trajectories and found that persistently low MPP was associated with higher 90-day mortality [39]. In a large MIMIC-IV–based study of sepsis-associated AKI, Fang Y et al. identified distinct dynamic MPP trajectories using group-based trajectory modeling, demonstrating that patients with persistently low MPP (< 60 mmHg) had significantly higher 90-day mortality, while maintaining MPP > 60 mmHg was protective [39]. These findings highlight the importance of tracking perfusion-related indices over time, highlighting the importance of dynamic physiologic trajectories rather than isolated single-point measurements, and provide a quantitative framework for linking hemodynamic trajectories with renal outcomes. Other ventilatory dimensions, including respiratory rate, tidal volume, mechanical power, and oxygenation targets, may also influence renal vulnerability. However, compared with driving pressure and P_mean_-related metrics, evidence directly linking these parameters to AKI remains less mature and requires further investigation.

### Phenotype-based stratification and precision trials

Given the marked heterogeneity among mechanically ventilated patients, phenotype-based approaches may enable more accurate risk prediction and targeted therapeutic strategies. Unsupervised clustering of key ventilatory variables could delineate distinct respiratory mechanics phenotypes that correspond to varying susceptibilities to AKI. Integrating these phenotypes with the clinical context (e.g., ARDS vs. non-ARDS, ventilator-induced lung injury, infection, or sepsis) could facilitate the construction of an individualized “AKI risk portrait.” Such stratification has the potential to inform personalized ventilator management and serve as a framework for adaptive or precision clinical trials. Recent analyses of ARDS sub-phenotypes have further validated the feasibility and clinical relevance of this phenotypic approach [40].

### Causal inference: mediation and effect modification

A comprehensive understanding of ventilator-associated AKI necessitates the integration of ventilatory, hemodynamic, and renal signals. Future investigations should couple respiratory mechanics phenotyping with intermediate physiological mediators to establish a multimodal assessment framework linking mechanical stress to renal injury. This integrative approach would enable the concurrent evaluation of mechanical load, venous congestion, and renal microcirculatory stress, thereby delineating a quantitative pathway from ventilatory exposure to AKI progression. Advanced analytical methods, including causal mediation analysis and time-dependent modeling, could further quantify the proportion of AKI risk attributable to these mediating mechanisms [18]. Because ventilatory exposure, hemodynamic status, and AKI development evolve simultaneously, future studies should account for time-varying confounding using appropriate causal-inference approaches, including marginal structural models, joint modeling strategies, and time-dependent mediation analyses. AKI outcomes should also consider timing (early versus late AKI), trajectories, and competing events rather than relying solely on binary definitions.

### Integrative multimodal monitoring embedded in trials

To advance these research directions, future studies should adopt standardized and data-rich methodological frameworks. Harmonized collection of ventilatory, hemodynamic, and renal variables is essential to ensure reproducibility and enable cross-study comparisons. Prospective protocols should incorporate consistent definitions of early versus late AKI, prespecified causal diagrams, and analytical plans for mediation and effect-modification analyses to delineate mechanistic pathways linking ventilatory exposures to renal outcomes.

Multicenter collaboration will be critical to validate physiologic thresholds such as optimal driving pressure, mean airway pressure, and renal perfusion pressure, and to evaluate renal-protective ventilation strategies in pragmatic clinical trials. Integration of multimodal monitoring, encompassing respiratory mechanics phenotyping, Doppler-based renal indices, and organ perfusion imaging, should be embedded into trial design to capture real-time physiological feedback. Such rigorous and harmonized methodologies will accelerate translation from mechanistic insight to precision prevention of ventilator-associated AKI. Major challenges include establishing causal relationships, defining clinically actionable thresholds, separating ventilator-related effects from concurrent ICU exposures, and validating multimodal strategies in prospective trials.

### Frontiers in cross-organ monitoring

A promising frontier lies in cross-organ physiological monitoring, particularly the simultaneous assessment of cerebral and renal perfusion during mechanical ventilation. Integrating cerebral oximetry (NIRS), transcranial Doppler (TCD), and EEG-derived cortical activity with renal Doppler indices—such as the renal resistive index (RRI) and venous excess ultrasound (VExUS) score—may help identify “dual-organ vulnerability windows,” periods in which both the brain and kidney experience compromised perfusion. Incorporating advanced perfusion imaging modalities, including contrast-enhanced ultrasound (CEUS) and blood oxygen level–dependent magnetic resonance imaging (BOLD-MRI), could further facilitate spatiotemporal mapping of cortical and medullary renal blood flow, thereby bridging bedside physiological monitoring with mechanistic insight into organ cross-talk [18].

### Methodological limitations and evidence gaps

The current evidence base linking mechanical ventilation to AKI is constrained by important methodological limitations and persistent gaps. First, most studies are observational, limiting causal inference and leaving residual confounding related to disease severity, fluid management, vasoactive therapy, and nephrotoxic exposure. Differences in ventilatory practice across centers, as well as inconsistent time windows and sampling strategies for ventilatory exposures, further complicate exposure quantification and comparability across studies. In addition, mediation and effect-modification are likely present, positive fluid balance may partially mediate the association between elevated P_mean_ and AKI risk, whereas venous congestion and renal vascular resistance may operate as intermediate physiological variables. Without prespecified causal frameworks and formal mediation analyses, separating direct from indirect effects remains challenging. Likewise, effect modification by lung compliance, recruitability, or right ventricular function may alter how ΔP or PEEP influences renal outcomes, reinforcing the need for individualized evaluation. Temporal heterogeneity in AKI presentation (early vs. late onset) is also frequently overlooked, although late AKI may better reflect the causal impact of ventilatory exposure.

In addition, definitions and operationalization of exposure variables (ΔP, P_mean_, PEEP) and renal outcomes (AKI criteria, timing, and staging) vary widely across studies, limiting cross-study synthesis. Publication bias and selective reporting may also influence the apparent consistency of associations. Experimental studies, while informative, are often small and short-term, which may limit extrapolation to heterogeneous ICU populations.

Beyond these limitations, major evidence gaps remain. Large prospective studies and randomized trials using AKI as a primary endpoint are scarce. Physiologic and imaging-based tools (e.g., RRI and VExUS), while promising for bedside assessment of renal hemodynamics, have largely been evaluated in small, single-center cohorts and require multicenter validation. Biomarkers, such as urinary NGAL and TIMP-2·IGFBP7 have demonstrated predictive potential across multiple clinical cohorts; however, their clinical utility remains context-dependent, particularly when integrated with ventilatory and hemodynamic variables in multimodal prediction models. Finally, much of the current literature is concentrated in ARDS and COVID-19 cohorts, with comparatively limited evidence in other critical conditions such as sepsis, heart failure, and major surgery. Addressing these gaps through harmonized data collection, standardized definitions, and causally structured analyses will be essential to translate mechanistic insights into kidney-protective ventilation strategies.

## Conclusions

Mechanical ventilation–associated AKI is a multifactorial syndrome arising from a complex interplay between respiratory mechanics, hemodynamic perturbations, venous congestion, inflammation, and metabolic stress. Current evidence suggests that renal risk is influenced not only by static ventilatory settings, but also by the temporal trajectories of ventilatory and perfusion-related variables. Integrating ventilatory parameters with multimodal bedside assessment of renal perfusion and congestion may improve early recognition of high-risk patients and support individualized kidney-protective ventilation strategies. Future research should prioritize standardized physiologic monitoring, causally structured analyses, and prospective multicenter studies to translate mechanistic insights into precision critical care interventions.

### Clinical take-home message

Mechanical ventilation–associated AKI should be understood as a dynamic manifestation of lung–kidney cross-talk rather than the isolated effect of individual ventilatory parameters. Bedside assessment should integrate ventilatory exposure (ΔP, PEEP, Pmean), systemic and renal perfusion, venous congestion, and early kidney injury markers, supporting kidney-protective ventilation strategies based on individualized adjustment of ventilatory and hemodynamic targets to preserve effective renal perfusion while minimizing congestion and inflammatory stress.

### Tweet

MV-associated AKI reflects dynamic lung-kidney cross-talk; multimodal monitoring may support kidney-protective ventilation.

## Data Availability

Not applicable. This narrative review did not generate or analyze new datasets.
